# Estimated pulse wave velocity is associated with all-cause and cardiovascular mortality in individuals with stroke: A national-based prospective cohort study

**DOI:** 10.1097/MD.0000000000041608

**Published:** 2025-02-14

**Authors:** Jiazheng Li, Cheng Jiang, Jialiang Ma, Feng Bai, Xulong Yang, Qi Zou, Peng Chang

**Affiliations:** a Department of Cardiology, Lanzhou University Second Hospital, Lanzhou, China.

**Keywords:** all-cause mortality, arterial stiffness, cardiovascular disease mortality, cohort study, estimated pulse wave velocity, Framingham Risk Score (FRS), mortality prediction, National Health and Nutrition Examination Survey (NHANES), risk assessment models, stroke

## Abstract

Extensive evidence underscores the potential of estimated pulse wave velocity (ePWV) as a robust tool for predicting disease prevalence and mortality. However, its comparative effectiveness in forecasting all-cause and cardiovascular disease (CVD) mortality, particularly among stroke populations, remains inadequately characterized in relation to the traditional Framingham Risk Score (FRS) model. This prospective study included 1202 individuals with stroke from the National Health and Nutrition Examination Survey conducted between 1999 and 2014, with comprehensive follow-up data. Survey-weighted Cox regression models were employed to examine the association between ePWV and the risks of all-cause and CVD mortality. Subgroup analyses were performed to evaluate the stability of ePWV in predicting these outcomes. A generalized additive model was utilized to explore the dose–response relationship between ePWV and mortality risk. Receiver operating characteristic curves were then used to assess and compare the prognostic capabilities of ePWV and FRS models for 10-year all-cause and CVD mortality. After adjustment for relevant covariates, each 1 m/s increase in ePWV was associated with a 44% and 65% increase in all-cause and CVD mortality, respectively. ePWV demonstrated consistent prognostic performance across the majority of stroke subpopulations. Notably, ePWV exhibited a nonlinear relationship with all-cause mortality (*P* for nonlinearity = .045) while maintaining a linear association with CVD mortality (*P* for nonlinearity = .293). Furthermore, ePWV outperformed the FRS model in predicting 10-year all-cause (Integrated Discrimination Improvement = 0.061, 95% confidence interval: 0.031–0.095, *P* = .007) and CVD mortality (95% confidence interval: 0.005–0.083, *P* = .02). ePWV is an independent risk factor for both all-cause and CVD mortality in individuals with stroke, demonstrating superior predictive value compared to the traditional FRS model for forecasting these outcomes.

## 1. Introduction

Stroke, a leading cerebrovascular disorder, remains a significant cause of neurological disability in both adults and children, posing a major global health challenge. A 2019 report identified stroke as the second leading cause of death, underscoring its role as a major risk factor for disability.^[1]^ Ischemic stroke, the predominant stroke subtype, accounts for the majority of cases, with over 50% attributed to cerebrovascular atherosclerotic plaques. The rupture of these plaques and the resulting arterial stiffness are key drivers in the progression of vascular lesions.^[2]^ Epidemiological data indicate that approximately 13.7 million stroke events occur annually worldwide, leading to 5.5 million deaths.^[3]^ By 2050, stroke-related mortality is projected to reach 9.7 million.^[4]^ In addition, many stroke survivors endure prolonged rehabilitation, placing substantial strain on both families and healthcare systems. The societal and economic burdens are considerable, highlighting the urgent need for effective prevention and management strategies.^[5]^ Stroke prevention is further complicated by a combination of modifiable and non-modifiable risk factors, including hypertension, obesity, air pollution, diabetes, smoking, and aging, necessitating enhanced risk assessment tools.^[1]^

Arterial stiffness is now universally recognized as a critical factor in stroke progression.^[6,7]^ Prior research has identified arterial stiffness as an independent risk factor for both stroke prevalence and mortality.^[7,8]^ Characterized by diminished arterial elasticity, arterial stiffness is typically exacerbated by aging, hypertension, and metabolic disorders. Increased stiffness leads to elevated cardiac workload and impaired cerebrovascular autoregulation, both of which are central to the pathophysiology of stroke.^[9]^

The estimated pulse wave velocity (ePWV), a noninvasive parameter integrating age and blood pressure, serves as an excellent marker for assessing arterial stiffness. Growing evidence supports ePWV as a reliable indicator for predicting all-cause and cardiovascular disease (CVD) mortality at the population level.^[10,11]^ Recent studies have demonstrated that each one-unit increase in ePWV corresponds to a 53% (hazard ratio [HR]: 1.53, 95% confidence interval [CI]: 1.23–1.90) increased risk for total stroke, a 42% (HR: 1.42, 95% CI: 1.11–1.83) increased risk for ischemic stroke, and a 92% (HR: 1.92, 95% CI: 1.21–3.03) increased risk for hemorrhagic stroke.^[12]^ Despite these findings, there remains a critical gap in understanding the utility of ePWV for predicting outcomes in patients with stroke, where vascular health is already compromised. Assessing the predictive value of ePWV in this specific context could provide valuable insights for patient management and enhance prognostic accuracy.

The Framingham Risk Score (FRS) has long served as a standard tool for estimating CVD risk.^[13]^ However, its predictive ability may be limited in certain populations, such as individuals with preexisting stroke.^[14]^ While the role of ePWV in predicting CVD events has been examined in various cohorts, its association with mortality in patients with stroke, and its comparative efficacy relative to the traditional FRS model, remain unclear. To address this gap, the present study evaluates the predictive potential of ePWV for all-cause and CVD mortality within the stroke cohort from the National Health and Nutrition Examination Survey (NHANES) spanning 1999 to 2014. This study is the first to specifically investigate ePWV’s ability to predict both all-cause and CVD mortality in patients with stroke and to directly compare its predictive performance with that of the FRS. Our findings offer a novel approach to refining risk stratification and improving clinical prognostication for patients with stroke.

## 2. Methods

### 2.1. Study design and population

This cohort study utilized data from the NHANES, covering the period from 1999 to 2014, with follow-up extending through December 31, 2019. Detailed information on the sampling methods and data collection process is available on the NHANES website (http://www.cdc.gov/nchs/nhanes.htm). All participants in NHANES provided explicit written informed consent, and the study protocol was approved by the US National Center for Health Statistics Research Ethics Review Board.^[15]^

A total of 78,518 individuals were enrolled in NHANES between 1999 and 2014, of whom 1631 stroke-diagnosed participants aged over 18 years were identified. This study excluded participants with a history of cancer (309), those without ePWV data (117), those with missing follow-up information (2), or those who were pregnant (1). The final dataset for this analysis included 1202 patients with stroke. The study was conducted in compliance with the STROBE guidelines.

### 2.2. Diagnosis of stroke

Stroke diagnoses were based on self-reported medical history, as indicated in the medical conditions section of NHANES. Participants were asked, “Has a physician or other health professional ever told you that you had a stroke?” Those who answered affirmatively were classified as having a history of stroke.

### 2.3. Evaluation of ePWV

ePWV was calculated using an established algorithm^[16]^ derived from the Reference Values for Arterial Stiffness Collaboration:^[17]^


$$\begin{array}{l} \text{ePWV}=9.587-(0.402\times \text{age})+\left[4.560\times 0.001\times \left({\text{age}}^{2}\right)\right]\\ -\left[2.621\times 0.00001\times \left({\text{age}}^{2}\right)\times \text{MBP}\right]+\left(3.176\times 0.001\times \text{age}\times \text{MBP}\right)\\ -\left(1.832\times 0.01\times \text{MBP}\right). \end{array}$$

In this formula, age is expressed in years, and mean blood pressure is calculated as diastolic blood pressure + 0.4 × [systolic blood pressure − diastolic blood pressure].^[18]^ Blood pressure was measured using a standard sphygmomanometer after participants sat quietly for 5 minutes. Measurements were performed by trained personnel, and the reported blood pressure value was the average of at least 3 separate readings.

### 2.4. Study endpoints

The primary outcomes of this study were all-cause mortality and CVD mortality. All-cause mortality included deaths from any cause, while CVD mortality was specifically defined as deaths attributed to cardiovascular disease, classified according to the International Classification of Diseases, 10th Revision codes (I00–I09, I11, I13, I20–I51).

### 2.5. Assessment of other variables

Demographic data, including age, sex, race, body mass index (BMI), education level, family income, lifestyle factors such as smoking and drinking habits, medical history, and medication use, were collected via standardized questionnaires administered during family interviews and mobile examination center visits. Biochemical indicators were evaluated following a strict protocol, the details of which are outlined in the NHANES Laboratory/Medical Technician Procedure Manual.^[19]^ Additionally, each participant’s traditional risk model (FRS) was assessed.

For ease of data integration, several variables were categorized: race (non-Hispanic White, non-Hispanic Black, Mexican American, or other); education level (<9th grade, 9–11th grade/high school graduate or equivalent, college graduate or above). Smoking status was categorized as never (fewer than 100 cigarettes lifetime), former (more than 100 cigarettes lifetime, currently not smoking), and current (more than 100 cigarettes lifetime, currently smoking either daily or occasionally). Alcohol consumption was classified as never (fewer than 12 drinks lifetime), former (more than 12 drinks lifetime, not in the past year), mild/moderate (up to 1 drink per day for women or up to 2 drinks per day for men in the past year), and heavy (more than 1 drink per day for women or more than 2 drinks per day for men in the past year).

### 2.6. Statistical analysis

Appropriate statistical weights were applied for the analysis. Continuous baseline characteristics were reported as weighted means with standard errors, while categorical variables were presented as unweighted counts with weighted percentages. HRs and 95% CIs for the association between ePWV and all-cause and CVD mortality were calculated using survey-weighted Cox regression models. The variance inflation factor was employed to assess multicollinearity among variables, and variables with a variance inflation factor ≥ 10 were excluded to reduce overfitting.^[20]^ All baseline variables were treated as potential predictors in the multiple regression model, with confounding covariates systematically included.

Subgroup analyses were conducted based on clinical characteristics, including sex (male, female), age (< 65 years, ≥65 years), BMI (< 30 kg/m^2^, ≥30 kg/m^2^), race (non-Hispanic White, non-Hispanic Black, Mexican American, other), and history of chronic disease. Interaction *P*-values were also calculated. To explore the dose–response relationship between ePWV and mortality risk, a generalized additive model was employed.^[21]^ The log-likelihood ratio test was used to determine *P*-values for nonlinearity. For nonlinear associations, a two-piecewise linear regression model was applied to identify the inflection point at which the ePWV-mortality ratio significantly changed on the smoothing curve.^[22]^ A time-dependent receiver operating characteristic (ROC) curve was constructed to assess the predictive accuracy of ePWV for 10-year all-cause and CVD mortality. Harrell C-statistic (C-index) was used to compare the predictive performance of the ePWV and FRS models, evaluating the discriminatory power of these models for all-cause and CVD mortality in patients with stroke. The Integrated Discrimination Improvement index quantified the differential predictive value of the ePWV and FRS models for both all-cause and CVD mortality in patients with stroke.^[23]^

All statistical analyses were performed using R software (http://www.R-project.org, The R Foundation) and EmpowerStats (Version 4.2.0, www.R-project.org, X&Y Solutions, Inc., Boston, MA). A *P*-value of <.05 was considered statistically significant.

## 3. Results

### 3.1. Demographics and baseline characteristics

A total of 1202 stroke participants were included in this study out of 78,518 enrolled between 1999 and 2014. The median follow-up period was 97 months (interquartile range: 62.3–139 months), during which 631 deaths were recorded, including 201 attributed to CVD. Table 1 presents the survey-weighted baseline characteristics of patients with stroke, representing 4,590,348 individuals. Of these, 43.62% were male, the mean age was 62.79 years, and the mean ePWV was 10.11 m/s. Notably, the mean BMI and systolic blood pressure were 29.81 kg/m^2^ and 131.01 mm Hg, respectively, indicating elevated levels above optimal thresholds.

**Table 1:** Survey-weighted baseline characteristics of the stroke patients from NHANES 1999 to 2014 (N = 1202, representing 4,590,348 individuals).

ePWV	10.11 (0.08)
Demographic	
Age (years)	62.79 (0.57)
Men	594 (43.62)
Race	
Non-Hispanic White	581 (69.25)
Non-Hispanic Black	330 (15.87)
Mexican American	170 (5.34)
Other races	121 (9.55)
Poverty income ratio	2.33 (0.06)
Parameters	
Body mass index	29.81 (0.25)
Systolic blood pressure	131.01 (0.82)
Diastolic blood pressure	69.59 (0.49)
Estimated glomerular filtration rate	74.14 (0.98)
Total cholesterol	5.05 (0.05)
High-density lipoprotein cholesterol	1.31 (0.02)
History of diseases	
Cardiovascular heart diseases	188 (16.50)
Congestive heart disease	196 (15.51)
Heart attack	264 (20.51)
Diabetes mellitus	460 (33.68)
Hypertension	974 (79.44)
Hyperlipidemia	1002 (82.19)
Medication	
Antihypertensives	211 (17.81)
Glucose-lowering drugs	64 (6.04)
Lipid-lowering drugs	114 (9.83)
Lifestyle	
Smoking	
Never	505 (41.93)
Former	409 (31.23)
Current	288 (26.84)
Drinking	
Never	205 (17.54)
Former	446 (36.50)
Mild/Moderate	262 (26)
Heavy	193 (19.96)
Framingham Risk Score	13.79 (0.0.25)

Continuous variables are expressed as weighted mean (Standard error, SE).

Categorical variables are expressed as counts (weighted %).

ePWV = estimated pulse wave velocity, NHANES = National Health and Nutrition Examination Survey.

### 3.2. Association between ePWV and all-cause and CVD mortality

Weighted univariable and multivariable Cox regression analyses were conducted to assess the association between ePWV and both all-cause and CVD mortality (Table 2). In the unadjusted model, each 1 m/s increase in ePWV was associated with a 41% increase in all-cause mortality (hazard ratio [HR] 1.41, 95% CI: 1.34–1.48; *P* < .001) and a 39% increase in CVD mortality (HR 1.39, 95% CI: 1.29–1.49; *P* < .001). Further analysis using multivariate Cox regression revealed that ePWV was an independent risk factor for both all-cause and CVD mortality. Each 1 m/s increase in ePWV was associated with a 44% to 67% increase in all-cause mortality and a 51% to 73% increase in CVD mortality across 5 models. Even after adding the FRS to model 6, the results remained robust.

**Table 2:** Survey-weighted Cox proportional hazard results examining the association of ePWV on all-cause and CVD mortality in stroke patients from NHANES 1999 to 2014.

ePWV, 1 m/s increase	Death	Unadjusted model	Adjusted Model 1	Adjusted Model 2	Adjusted Model 3	Adjusted Model 4	Adjusted Model 5	Adjusted Model 6
		HR (95%CI)	HR (95%CI)	HR (95%CI)	HR (95%CI)	HR (95%CI)	HR (95%CI)	HR (95%CI)
All-cause mortality	631	1.41 (1.34–1.48)*	1.50 (1.40–1.60)*	1.44 (1.31–1.57)*	1.67 (1.63–1.72)*	1.46 (1.33–1.60)*	1.51 (1.37–1.67)*	1.44 (1.29–1.61)*
CVD mortality	201	1.39 (1.29–1.49)*	1.51 (1.37–1.67)*	1.54 (1.33–1.78)*	1.73 (1.64–1.82)*	1.57 (1.35–1.82)*	1.67 (1.41–2.0)*	1.65 (1.38–1.98)*

Model 1 adjust systolic blood pressure, diastolic blood pressure.

Model 2 adjusted Model 1 plus other demographic variables including gender, race (non-Hispanic White, non-Hispanic Black, Mexican American, and other races) and poverty income ratio (continuous).

Model 3 adjusted Model 2 plus other parameters including body mass index (continuous), estimated glomerular filtration rate (continuous), total cholesterol (continuous), and high-density lipoprotein cholesterol (continuous).

Model 4 adjusted Model 3 plus history of diseases including cardiovascular heart diseases (yes/no), congestive heart disease (yes/no), diabetes mellitus (yes/no), heart attack (yes/no), hypertension (yes/no), and hyperlipidemia (yes/no).

Model 5 adjusted Model 4 plus medication including antihypertensives (yes/no), glucose-lowering drugs (yes/no), lipid-lowering drugs (yes/no), smoking (never, former, and current), and drinking (never, former, mild/moderate, and heavy).

Model 6 adjusted Model plus Framingham Risk Score.

* *P*-value < .001

### 3.3. Subgroup analysis

**Figure 1.**
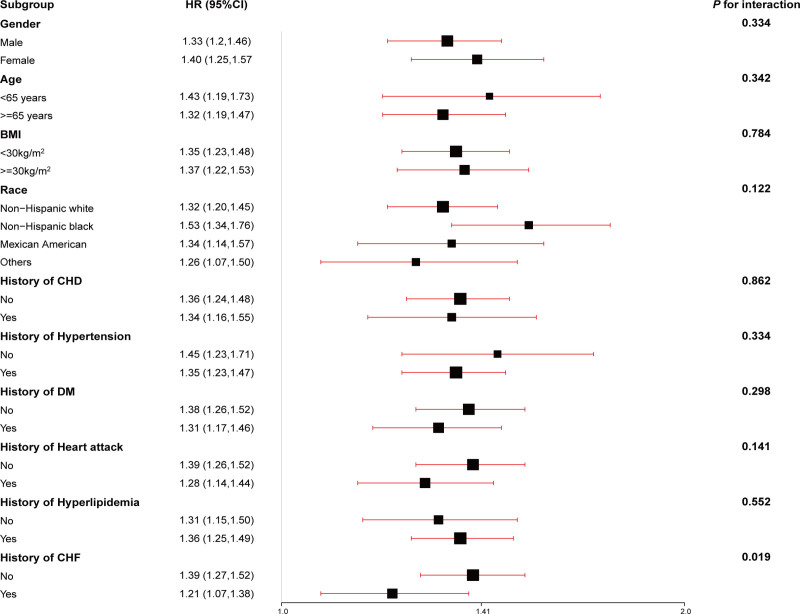
Association between ePWV and all-cause mortality in stroke subgroups. The model was adjusted by the following variables: systolic blood pressure, diastolic blood pressure, gender, race, poverty income ratio, body mass index, estimated glomerular filtration rate, total cholesterol, high-density lipoprotein cholesterol, cardiovascular heart diseases, congestive heart disease, diabetes mellitus, heart attack, hypertension, hyperlipidemia, antihypertensives, glucose-lowering drugs, lipid-lowering drugs, smoking, drinking and Framingham Risk Score. ePWV = estimated pulse wave velocity.

**Figure 2.**
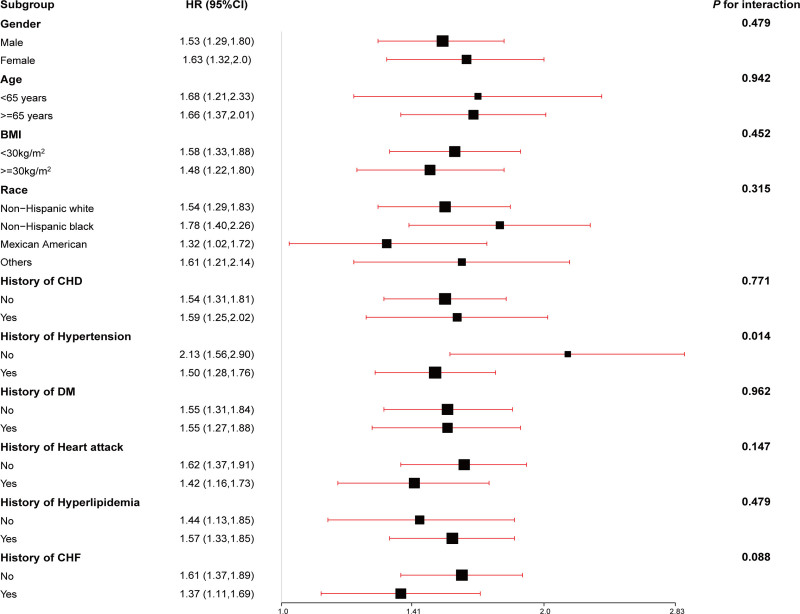
Association between ePWV and CVD mortality in stroke subgroups. The model was adjusted systolic blood pressure, diastolic blood pressure, gender, race, poverty income ratio (PIR), body mass index (BMI), estimated glomerular filtration rate, total cholesterol (TC), high-density lipoprotein cholesterol (HDL-C), cardiovascular heart diseases, congestive heart disease, diabetes mellitus (DM), heart attack, hypertension, hyperlipidemia, antihypertensives, glucose-lowering drugs, lipid-lowering drugs, smoking, drinking and Framingham Risk Score. CVD = cardiovascular disease, ePWV = estimated pulse wave velocity.

Subgroup analyses were performed to evaluate the robustness of the association between ePWV levels and both all-cause and CVD mortality (Figs. 1 and 2). The relationship between ePWV and all-cause mortality remained highly consistent across most subgroups, including gender (*P* = .334), age (*P* = .342), BMI (*P* = .784), race (*P* = .122), history of coronary heart disease (CHD; *P* = .862), hypertension (*P* = .334), diabetes mellitus (DM; *P* = .298), heart attack (*P* = .141), and hyperlipidemia (*P* = .552). However, participants with a history of congestive heart failure (CHF) exhibited a lower risk of all-cause mortality (HR 1.21, 95% CI: 1.07–1.38 vs HR 1.39, 95% CI: 1.27–1.52; *P* = .019) compared to those without a history of CHF.

Similarly, the association between ePWV and CVD mortality demonstrated consistent performance across subgroups, including gender (*P* = .479), age (*P* = .942), BMI (*P* = .452), race (*P* = .315), history of CHD (*P* = .771), DM (*P* = .962), heart attack (*P* = .147), hyperlipidemia (*P* = .479), and CHF (*P* = .088). Notably, participants with a history of hypertension showed a lower risk of CVD mortality (HR 1.50, 95% CI: 1.28–1.76 vs HR 2.13, 95% CI: 1.58–2.90; *P* = .014) compared to those without a hypertension history.

### 3.4. Dose–response association between ePWV and mortality risk

Table 3 presents the results from a two-piecewise linear regression model, illustrating the relationship between ePWV and the risk of all-cause and CVD mortality among patients with stroke, as derived from Model 5 with maximum adjustment for covariates. A positive, nonlinear association was identified between ePWV and all-cause mortality (*P* for nonlinearity = .045). Two inflection points for ePWV were determined. A 1 m/s increase in ePWV was associated with a 48% increase in all-cause mortality (HR = 1.48, 95% CI: 1.16–1.89, *P* = .002) when ePWV values were <13.25 m/s. A more pronounced positive correlation emerged when ePWV values exceeded 13.25 m/s (HR = 4.16, 95% CI: 1.23–14.08, *P* = .002). In contrast, ePWV and CVD mortality demonstrated a positive linear correlation (*P* for nonlinearity = .293), with each 1 m/s increase in ePWV correlating to a 39% higher risk of CVD mortality (HR = 1.39, 95% CI: 1.11–1.73, *P* = .004). The dose–response relationship between ePWV and mortality risk is depicted in Figure S1A, Supplemental Digital Content, http://links.lww.com/MD/O404 (all-cause mortality) and Figure S1B, Supplemental Digital Content, http://links.lww.com/MD/O404 (CVD mortality).

**Table 3:** The results of two-piecewise linear regression model for ePWV and the risk of all-cause and CVD mortality in stroke patients.

Outcome	Inflection point of ePWV (m/s)	HR	95% CI	*P*-value	*P* for nonlinear
All-cause mortality	<13.25	1.48	1.16–1.89	.002	.045
	≥13.25	4.16	1.23–14.08	.022	
CVD mortality		1.39	1.11–1.73	.004	.293

HRs have been adjusted by the following variables: systolic blood pressure, diastolic blood pressure, gender, race, poverty income ratio, body mass index, estimated glomerular filtration rate, total cholesterol, high-density lipoprotein cholesterol, cardiovascular heart diseases, congestive heart disease, diabetes mellitus, heart attack, hypertension, hyperlipidemia, antihypertensives, glucose-lowering drugs, lipid-lowering drugs, smoking, drinking, and Framingham Risk Score.

### 3.5. Predictive performance of ePWV for all-cause and CVD mortality

**Figure 3.**
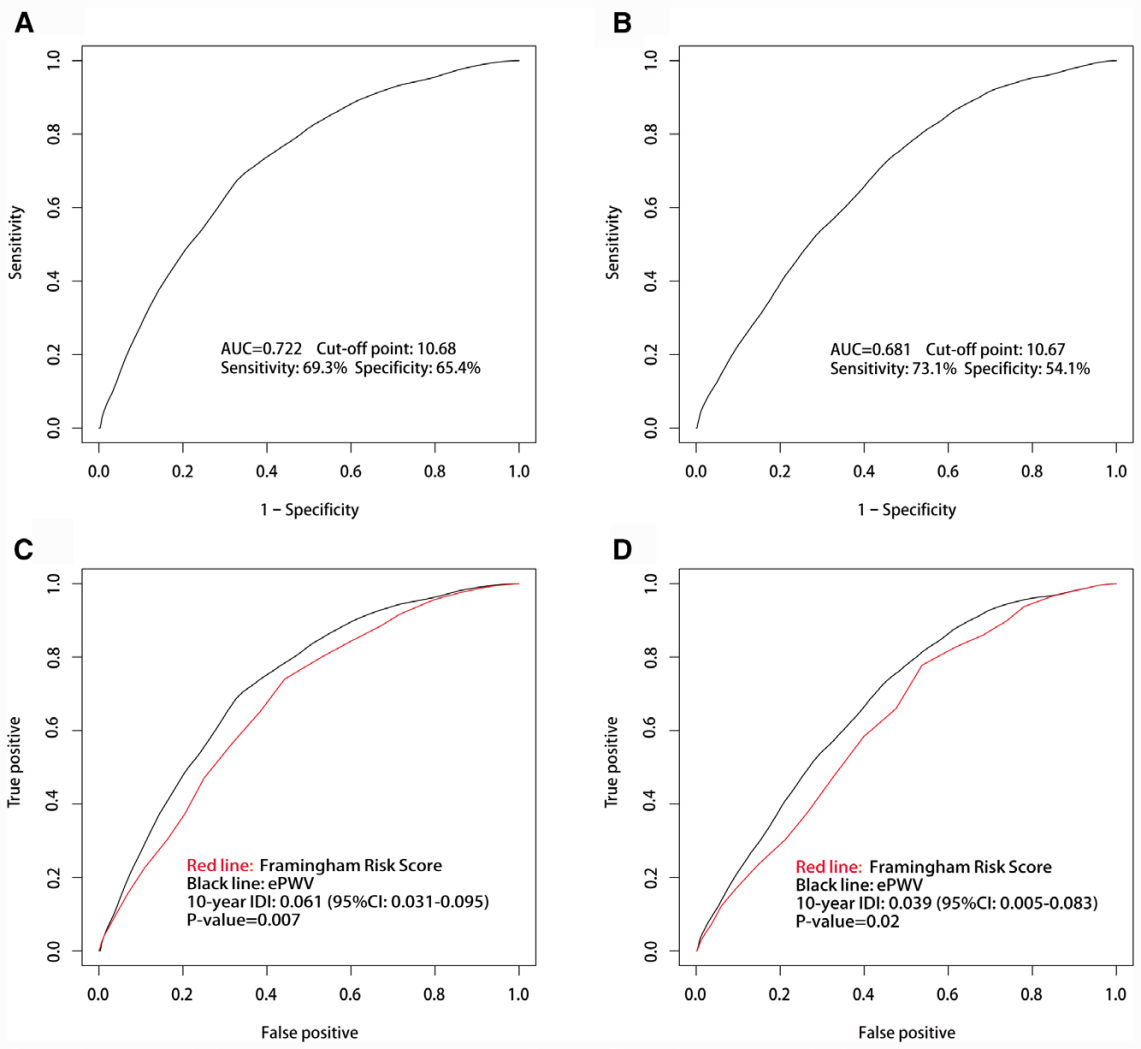
Receiver operating characteristics (ROC) curves of ePWV in 10-year all-cause mortality and CVD mortality. (A) ROC curve of ePWV in 10-year all-cause mortality. (B) ROC curve of ePWV in 10-year CVD mortality. (C) ROC curves of ePWV and FRS in 10-year all-cause mortality. (D) ROC curves of ePWV and FRS in 10-year CVD mortality. CVD = cardiovascular disease, ePWV = estimated pulse wave velocity.

ROC curves were employed to assess the predictive capacity of ePWV for 10-year all-cause and CVD mortality (Fig. 3). ePWV consistently predicted 10-year all-cause mortality with an area under the curve of 0.722, at a cutoff value of 10.68 m/s, with sensitivity and specificity of 69.3% and 65.4%, respectively (Fig. 3A). Similarly, ePWV demonstrated reasonable predictive accuracy for CVD mortality (area under the curve = 0.681), with sensitivity and specificity of 73.1% and 54.1%, respectively, at a cutoff of 10.67 m/s (Fig. 3B), which closely matched the cutoff for all-cause mortality. A comparison of the predictive performance of ePWV and the FRS model for 10-year all-cause and CVD mortality is shown in Figure 3C and D. ePWV outperformed the FRS model in both all-cause mortality (Fig. 3C) and CVD mortality (Fig. 3D). The 10-year Integrated Discrimination Improvement was 0.061 for all-cause mortality (95% CI: 0.031–0.095, *P* = .007) and 0.039 for CVD mortality (95% CI: 0.005–0.083, *P* = .02). These results suggest that ePWV was 6.1% and 3.9% more effective than the FRS model in predicting all-cause and CVD mortality, respectively.

## 4. Discussion

In this large, long-term cohort study, 1206 patients with stroke from NHANES were enrolled, revealing a positive correlation between ePWV and an increased risk of both all-cause and CVD mortality over a 10-year follow-up period. A 1 m/s increase in ePWV was associated with a 51% rise in all-cause mortality and a 67% escalation in CVD mortality. Furthermore, a nonlinear relationship was observed between ePWV and all-cause mortality, while a linear association was identified with CVD mortality. Notably, ePWV demonstrated superior predictive power for 10-year all-cause and CVD mortality when compared to the FRS model.

Previous studies have explored the relationship between ePWV and mortality. Prelević et al found that each 1 m/s increment in ePWV led to a 15% increase in CVD mortality risk (HR: 1.15, 95% CI: 1.01–1.30), and a 90% increase in all-cause mortality risk (HR: 1.90, 95% CI: 1.28–2.82).^[24]^ In contrast, a study involving healthy individuals across 38 cohorts from 11 countries suggested that ePWV was associated with all-cause mortality (HR: 1.15, 95% CI: 1.08–1.22), but not with CVD mortality (HR: 0.97, 95% CI: 0.91–1.03) after adjusting for CVD risk factors.^[25]^ Other studies in obese and diabetic populations have consistently highlighted ePWV as a significant predictor of both all-cause and CVD mortality,^[10,26]^ underscoring its utility in populations with chronic health conditions.

However, research focusing specifically on ePWV in stroke populations remains limited. Previous work has indicated that ePWV is associated with increased stroke event risk (HR: 2.41, 95% CI: 1.66–3.53) and higher all-cause and cardio-cerebrovascular disease mortality in patients with stroke.^[27,28]^ The current study confirms that ePWV significantly elevates the risk of all-cause and CVD mortality in a large, long-term cohort of patients with stroke, with superior predictive performance compared to the classical FRS model for 10-year all-cause and CVD mortality.

The mechanisms by which ePWV influences stroke prevalence and mortality remain complex and are not fully elucidated. Aging, a key factor in both stroke and cardiovascular disease, contributes to cellular senescence, characterized by irreversible growth arrest, functional decline, and persistent low-grade inflammation.^[29–32]^ Increasing evidence suggests that the accumulation of senescent cells in the vasculature accelerates vascular aging,^[33]^ with these senescent cells producing reactive oxygen species, inflammatory cytokines, and chemokines, which impair nitric oxide bioavailability and contribute to vascular endothelial dysfunction and arterial stiffness.^[34,35]^ Additionally, the release of pro-inflammatory cytokines and cytotoxic molecules from senescent T cells exacerbates immune senescence, further promoting the interaction between vascular stiffness and oxidative stress.^[36,37]^

Moreover, arterial stiffness and the prevalence of hypertension are strongly correlated with aging.^[38]^ Epidemiological studies indicate that hypertension is twice as prevalent in the elderly population compared to younger individuals.^[39]^ Longitudinal studies have identified arterial stiffness, as measured by ePWV, as an independent predictor of adverse cardiovascular events, such as stroke and myocardial infarction, with ePWV serving as a reliable marker of arterial wall stiffness.^[11,40]^ This relationship highlights the potential of ePWV as a key biomarker for assessing stroke risk and mortality in this patient population.

The generalized additive model results revealed a nonlinear association between ePWV and the risk of all-cause mortality. The mechanisms underlying this nonlinear relationship remain unclear. All-cause mortality encompasses various causes of death, and the inherent heterogeneity among these causes may contribute to the observed nonlinearity with ePWV. Furthermore, the relationship between age and mortality risk is not always linear; especially in advanced age, the rate of progression of arterial stiffness may accelerate, indicating significant vascular deterioration and resulting in a sharp increase in mortality risk.

Subgroup analysis demonstrated that ePWV consistently performed well in predicting both all-cause and CVD mortality across most patient subgroups. Notably, significant differences were observed in the relationship between ePWV and mortality risk within the subgroups defined by the history of CHF and hypertension. Specifically, an increase in ePWV was more strongly associated with the risk of all-cause mortality in patients with stroke without a history of CHF (HR: 1.39 vs 1.21, *P* for interaction = .019). Similarly, in the hypertension subgroup, patients without a history of hypertension exhibited a higher risk for CVD mortality compared to those with hypertension (HR: 2.13 vs 1.5, *P* for interaction = .014). These results differ from those of a previous study.^[27]^ On the other hand, the association between ePWV and both all-cause and CVD mortality remained generally consistent across gender, age, BMI, race, and histories of CHD, DM, heart attack, and hyperlipidemia. Consequently, patients with stroke without a history of CHF and hypertension who demonstrate elevated ePWV may have an increased risk for all-cause and CVD mortality, respectively, necessitating enhanced monitoring and management.

Initially, the FRS model was employed as the standard tool for predicting CVD incidence, estimating the likelihood of a participant experiencing a cardiovascular event within ten years.^[41]^ Extensive evidence has underscored the superior predictive capability of the FRS in assessing CVD risk.^[42–44]^ The FRS model is widely used and has consistently demonstrated reliable performance in forecasting both all-cause and CVD mortality.^[45–47]^ In the current study, ePWV outperformed the FRS in predicting 10-year all-cause and CVD mortality, highlighting the potential utility of ePWV, particularly among patients with stroke. When comparing ePWV with the FRS model, the advantage of ePWV lies in its ability to account for the interactive effects of age and blood pressure, thereby offering a more comprehensive assessment of mortality risk. In contrast, the FRS relies on an additive approach to individual risk factors, which may fail to capture the synergistic influence between age and blood pressure. Furthermore, the FRS was primarily designed to assess cardiovascular event risk over a decade, rather than mortality risk, which may limit its accuracy in predicting long-term mortality outcomes. Age remains the strongest predictor of mortality risk. A recent study by Cheng et al demonstrated that ePWV outperformed the FRS in predicting mortality risk within both general and hypertensive populations, suggesting that ePWV may have broader applicability in mortality prediction.^[48,49]^ Consequently, as an easily measurable and accessible parameter, ePWV could serve a more critical role in dynamic risk management and long-term prognostic evaluation.

In conclusion, a positive correlation was observed between ePWV and both all-cause and CVD mortality, with increases in ePWV corresponding to heightened risks for both outcomes. Notably, a sharper increase in the risk of all-cause mortality was identified in patients with stroke exhibiting ePWV ≥ 13.25 m/s, indicating a threshold beyond which the risk escalates rapidly. These findings suggest that patients with stroke exhibiting elevated ePWV levels should be prioritized for targeted interventions to mitigate the risk of all-cause mortality.

### 4.1. Strengths and limitations

The strengths of this study lie in its large-scale, community-based stroke cohort, and the comprehensive long-term follow-up data. Dynamic models were developed to characterize the relationship between ePWV and both all-cause and CVD mortality, which may prove instrumental in estimating mortality risk within the stroke population. Moreover, the findings suggest that ePWV provides superior predictive value compared to the traditional FRS model.

However, several limitations must be acknowledged. First, the data are derived from a nationally representative sample of the United States, and the results may not be directly applicable to other populations. Consequently, the absence of external validation restricts the generalizability of these findings. Second, although the regression analysis adjusted for a wide range of covariates, the potential influence of unmeasured variables on the results cannot be excluded. Finally, due to the lack of a stratified analysis based on stroke subtypes, the specific significance of ePWV within each stroke subgroup could not be determined.

## 5. Conclusion

ePWV emerged as a significant independent risk factor for both all-cause and CVD mortality in patients with stroke, demonstrating enhanced predictive capability relative to traditional risk models. Notably, a nonlinear relationship between ePWV and all-cause mortality was observed, with a marked increase in risk when ePWV values exceeded 13.25 m/s, suggesting a threshold effect. This finding underscores the importance of intensive monitoring and timely intervention for patients with elevated ePWV. Incorporating ePWV into standard risk assessments could significantly refine prognostic tools, facilitating more accurate identification and management of high-risk patients with stroke, particularly those without conventional comorbidities such as CHF or hypertension.

## Acknowledgments

We thank Bullet Edits Limited for the linguistic editing and proofreading of the manuscript.

## Author contributions

**Conceptualization:** Jiazheng Li, Peng Chang.

**Formal analysis:** Jiazheng Li.

**Funding acquisition:** Jiazheng Li.

**Investigation:** Cheng Jiang.

**Validation:** Jialiang Ma, Peng Chang.

**Visualization:** Peng Chang.

**Writing – original draft:** Jiazheng Li.

**Writing – review & editing:** Jiazheng Li, Cheng Jiang, Jialiang Ma, Feng Bai, Xulong Yang, Qi Zou, Peng Chang.

## Supplementary Material


