# Supplementary material for: Estimated pulse wave velocity is associated with all-cause and cardiovascular mortality in individuals with stroke: A national-based prospective cohort study
Source: Medicine (Baltimore). 2025 Feb 14;104(7):e41608. doi: 10.1097/MD.0000000000041608 (PMC11835104; doi:10.1097/MD.0000000000041608)

Supplement

Figure S1. Smoothing curves of two-piecewise linear regression model for ePWV and the risk of all-cause (A) and CVD (B) mortality. The solid line and long dashed line represent the estimated odds ratio and its 95% confidence interval. ePWV, estimated pulse wave velocity.

HRs have been adjusted by the following variables: systolic blood pressure, diastolic blood pressure, gender, race, poverty income ratio, body mass index, estimated glomerular filtration rate, total cholesterol, high-density lipoprotein cholesterol, cardiovascular heart diseases, congestive heart disease, diabetes mellitus, heart attack, hypertension, hyperlipidemia, antihypertensives, glucose-lowering drugs, lipid-lowering drugs, smoking, drinking, Framingham Risk Score.

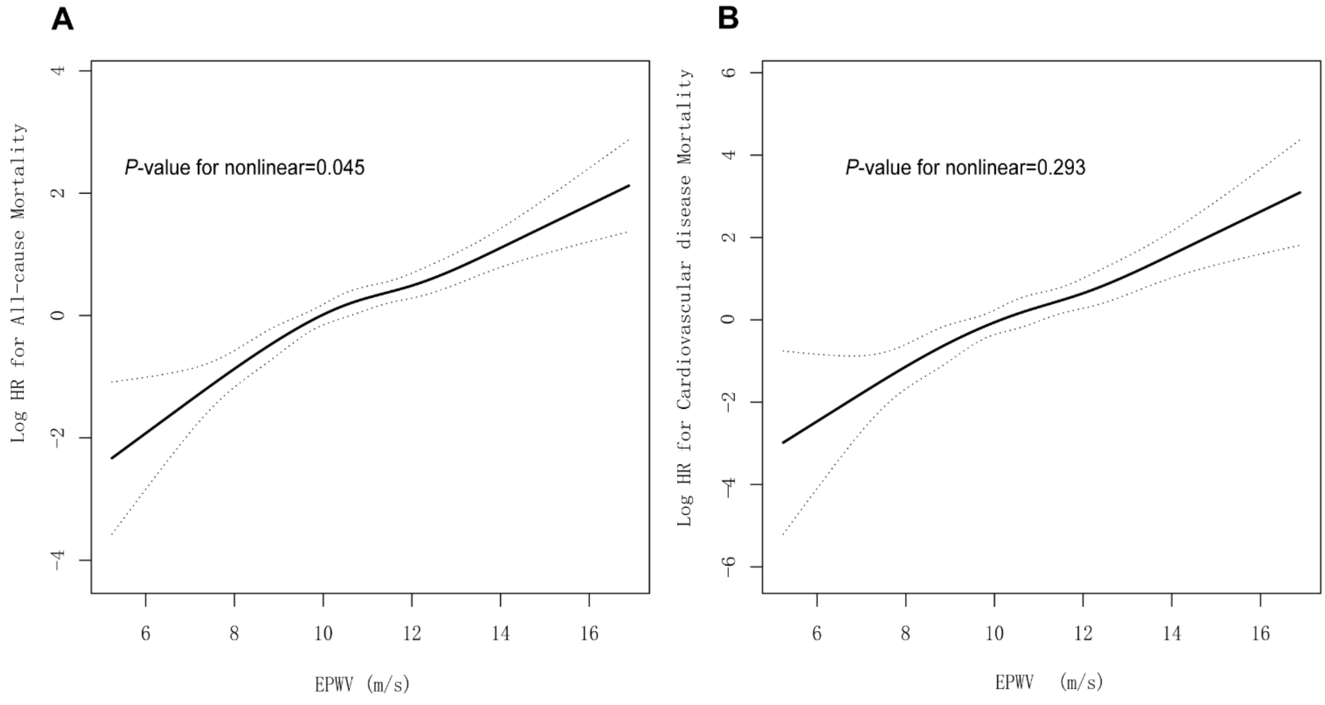

Supplement: Supplementary file 1 [file medi-104-e41608-s001.pdf]
